# Supplementary material for: Effect of Copolymer on the Wrinkle Structure Formation and Gloss of a Phase-Separated Ternary Free-Radical/Cationic Hybrid System for the Application of Self-Matting Coatings
Source: Polymers (Basel). 2022 Jun 11;14(12):2371. doi: 10.3390/polym14122371 (PMC9228514; doi:10.3390/polym14122371)
Supplement: Supplementary file 1 [file polymers-14-02371-s001.zip › polymers-1756362-supplementary.pdf]

polymers

Article

Effect of copolymer on the wrinkles structures formation and gloss of a phase-separated ternary free-radical/cationic hybrid system for self-matting UV-curable coating application

Ingrid Calvez [1], Caroline R. Szczepanski [2] and Véronic Landry [1,*]

[1] NSERC – Canlak Industrial Research Chair in interior Wood-Products finishes, Department of Wood and Forest Science, Université Laval, Québec, Canada; ingrid.calvez.1@ulaval.ca
[2] College of Engineering, Department of Chemical Engineering & Materials Science, Michigan State University, East Lansing, Michigan, USA; szcz@msu.edu
* Correspondence: veronic.landry@sbf.ulaval.ca; Tel.: +1-(418)-656-2131

Citation: Lastname, F.; Lastname, F.; Lastname, F. Title. *Polymers* **2022**, *14*, 2371. https://doi.org/10.3390/polym14122371

Academic Editor: Dalius Jucius

Received: 19 May 2022
Accepted: 6 June 2022
Published: 11 June 2022

Publisher's Note: MDPI stays neutral with regard to jurisdictional claims in published maps and institutional affiliations.

Copyright: © 2022 by the authors. Submitted for possible open access publication under the terms and conditions of the Creative Commons Attribution (CC BY) license (https://creativecommons.org/licenses/by/4.0/).

Supporting Information

Materials

Glycidyl methacrylate (GMA) was purchased from Sigma Aldrich (USA), n-butyl acrylate (nBA) from TCI (USA), N,N-Dimethylformanide (DMF) and methanol from Fisher Scientific (Thermo Fischer Scientific, USA), 2,2-azobisiobutyronitrile (AIBN) form MAT laboratories (Canada). All the chemicals were used as received.

Synthesis

According to Li et al., PBGMA was synthesized using a round bottom flask equipped with a mechanical stirrer and a condenser, nBA and GMA (0.4/0.1 mol/mol) were dissolved in DMF (150 mL) and 2 wt.% of AIBN was added, with respect to total weight of the monomers [1]. This initiator concentration was chosen to counteract the presence of inhibitor present in the initial monomers (monomethyl ether hydroquinone, <100 ppm). The copolymerization was carried out at 70 °C for 6h. The product was isolated by precipitation in a mixture of 1 methanol:1 deionized water and washed with deionized water. Finally, the copolymer was dried in a vacuum oven at 45 °C for 24h prior to use.
The copolymer structure of poly(butyl acrylate-co-glycidyl methacrylate) (PBGMA) is shown in Figure S1.

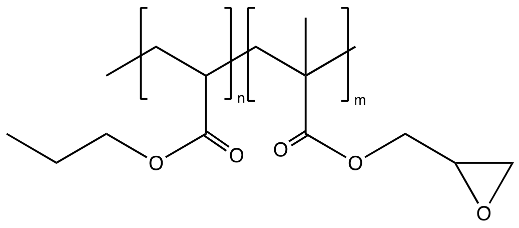

Figure S1. Chemical structure of poly(butyl acrylate-co-glycidyl methacrylate).

Characterizations and results

Infrared spectrum was obtained on a Fourier transform infrared spectrometer (Bruker – Invenio, USA) in the 4000– 400 cm^{-1} range. The molecular weights (Mn) and molecular weight distributions (Mw/Mn) of the sample were measured using gel permeation in refractive index (GPC, Waters 525) with two columns (PLgel 10 um MIXED-B LS 300 X 7,5mm, Agilent). Chloroform was used as the eluent at a flow rate of 1.0 $mL.min^{-1}$ and 100 µL per injection. Sample for the analysis was prepared as 2 $mg.mL^{-1}$ solutions in

chloroform and filtered through 0.45 mm Teflon filters prior to injection. Calibration curves were performed with 10 polystyrene standards with molecular weights between 580 and 3 152 000.

The epoxy value was titrated by HCl–acetone method. The HCl–acetone solution was prepared based on the volume ratio of HCl–acetone (2 mL HCl:30 mL acetone). About 0.5 g of the sample was dissolved in 20 mL of HCl–acetone solution by magnetic stirring. After complete dissolution, the phenolphthalein indicator were added, and the excess HCl was titrated by NaOH (1 mol.L^{-1}). The epoxy equivalent weight (EEW, g.mol^{-1}) was calculated by the following formula [2]:

$$EEW = \frac{(W \times 10)}{(V_0 - V) \times C} \times 100$$

where C is the concentration of NaOH standard solution (mol L^{-1}), V_0 is the blank consumption of NaOH standard solution (L), V is the sample consumption of NaOH standard solution (L), and W is the weight of the sample (g).

The FTIR spectra of the copolymer, n-BA and GMA are shown in Figure S2. The stretching vibration absorption peak of C=C double bond at 1630 cm^{-1} observed in the initial monomers n-BA and GMA, have disappeared in the copolymer spectrum. This result indicates that the two monomers of n-BA and GMA were converted into the copolymer. Furthermore, the peak at 760 cm^{-1} attributed to the epoxy group [3] and is observed before and after copolymerization.

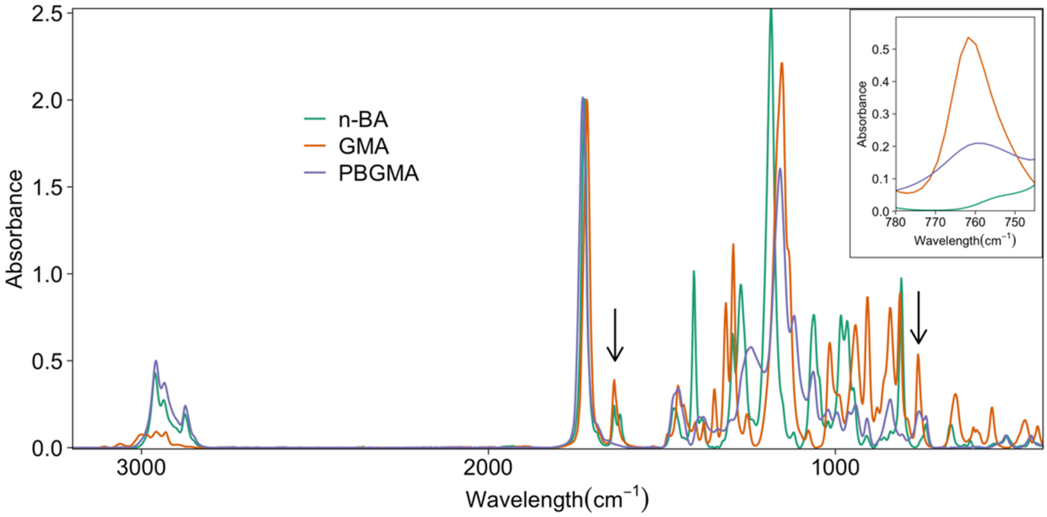

Figure S2. FTIR spectra of the n-BA, GMA and PBGMA.

The molecular weight (Mn) and epoxy equivalent value are presented in Table S1.

Table S1. Molecular weight and epoxy equivalent weight of PBGMA

	n-BA (mol)	GMA (mol)	Mn (g.mol^{-1})	Mw/Mn	EEW (g.mol^{-1})
PBGMA	0.40	0.10	10700	3.8	156

Design of experiments were performed to find the best mixture used in this study.

A ternary phase diagram was performed, and the experimental mixtures were presented in Table S2.

Table S2. Ternary design of experiments (DOEs) realized in this study. DOE 1 is the first diagram realized without limits, DOE 2 is the second diagram realized with limit X3=40 and supplementary points.

	**X1=CE**	**X2=PBDUA**	**X3=HDDMA**
DOE n°1			
**1**	100	0	0
**2**	0	100	0
**3**	0	0	100
**4**	50	50	0
**5**	0	50	50
**6**	50	0	50
**7**	33	33	33
**8**	16	16	66
**9**	16	66	16
**10**	66	16	16
DOE n°2			
**1**	60	0	40
**2**	10	10	80
**3**	0	30	70
**4**	0	60	40
**5**	30	30	40
**6**	30	0	70
**7**	10	40	50
**8**	60	40	0
**9**	20	20	60
**10**	40	10	50
Supplementary points			
**1**	50	15	35
**2**	40	15	45
**3**	64	4	32
**4**	67	4	29
**5**	5	80	15
**6**	14	73	14
**7**	17	70	13

Table S3. Post-cured conversion after 4 days of dark storage depending on PBGMA content by FTIR. Values are an average of 3 measures.

**PBGMA (wt.%)**	**EPOXY PART**		**(METH)ACRYLATE PART**	
	Top	Bottom	Top	Bottom
**0**	96 ± 2	98 ± 1	71 ± 2	75 ± 2
**1**	98 ± 1	97 ± 2	76 ± 2	74 ± 2
**2**	98 ± 0	98 ± 0	76 ± 2	79 ± 2
**3**	96 ± 2	98 ± 2	71 ± 2	78 ± 2
**5**	99 ± 0	98 ± 0	71 ± 2	79 ± 2
**7**	94 ± 3	93 ± 4	69 ± 2	71 ± 2
**10**	93 ± 2	92 ± 2	83 ± 2	82 ± 2

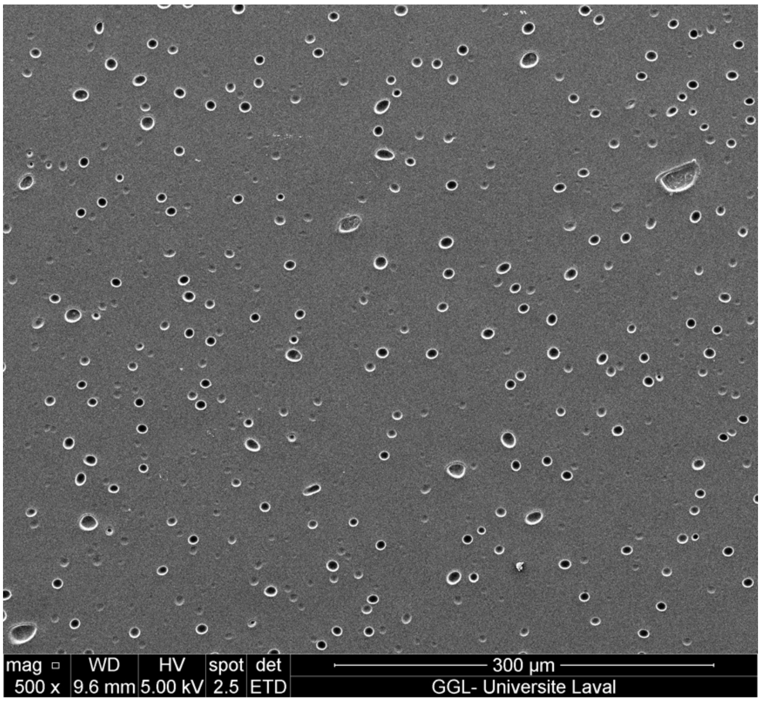

Figure S3. Surface analyses performed by SEM on reference system (without PBGMA). Circle pits have a diameter of approximately 12 µm.

Stress-strain curves were collected by DMA and are presented in Figure S4. The reference system showed a maximum stress of 34 MPa with approximately 8 % elongation at break. By adding 1 and 2 wt.% of PBGMA, elongation at break increases up to 11 %. The increased elongation at break could be due to the incorporation of PBGMA containing n-BA chains, which confers more flexibility to the network. With further increase of PBGMA, maximum stress and maximum strain tend to decrease. For example, with 10 wt.% of PBGMA, maximum stress is decreased by a factor of 2 compared to the reference. This decrease could be caused by adding PBGMA, which contributes to the flexibility of the system. Additionally, due to the significant decrease in the Young's modulus and elongation at break by adding more PBGMA, these systems can absorb less energy before breaking.

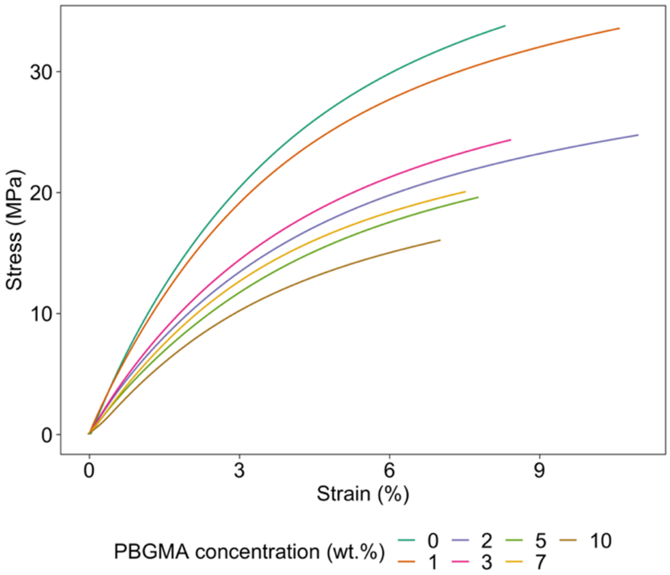

Figure S4. Stress and strain curves as a function of PBGMA concentration.

References

1. Li, X.; Ni, C.; Ma, F.; Yao, B.; Zhu, C. Preparation of Poly(N-Butyl Acrylate-Co-Glycidyl Methacrylate) and Its Application in Enhancement of Epoxy Resin. *Polym. Plast. Technol. Eng.* **2014**, *53*, 262–267, doi:10.1080/03602559.2013.844238.
2. Chen, L.; Zhang, C.; Du, Z.; Li, H.; Zhang, L.; Zou, W. Synthesis of Poly(n-Butyl Methacrylate)-(Glycidyl Methacrylate) Block Copolymer and Its Compatibilization at the Interface of the QD/Epoxy Nanocomposite for White LED Encapsulation †. **2015**, doi:10.1039/c5ra10197b.
3. Bakhshi, H.; Zohuriaan-Mehr, M.J.; Bouhendi, H.; Kabiri, K. Analysis Method Spectral and Chemical Determination of Copolymer Composition of Poly (Butyl Acrylate-Co-Glycidyl Methacrylate) from Emulsion Polymerization. *Polym. Test. 28*, 730–736, doi:10.1016/j.polymertesting.2009.06.003.
